# Supplementary material for: Demethylmenaquinone Methyl Transferase Is a Membrane Domain-Associated Protein Essential for Menaquinone Homeostasis in Mycobacterium smegmatis
Source: Front Microbiol. 2018 Dec 18;9:3145. doi: 10.3389/fmicb.2018.03145 (PMC6305584; doi:10.3389/fmicb.2018.03145)
Supplement: Supplementary file 1 [file Table_1.docx]

**Table S1**. Plasmids used in this study.

| **Plasmid name** | **Description** | **Selection** |
| --- | --- | --- |
| pMUM038 | Empty vector | Str |
| pMUM040 | Expression vector for *menA-HA* | Str |
| pMUM042 | Expression vector for *menG-HA* | Str |
| pMUM055 | Knockout construct to replace endogenous *menG* | Hyg/Suc |
| pMUM058 | Expression vector for *menG-mTurquoise-HA* | Str |
| pMUM087 | Empty vector | Kan |
| pMUM098 | Expression vector for *menG-HA* | Kan |
| pMUM119 | Expression vector for *menG-HA-DAS* | Str |
| pGMCT-3q-taq25 | Expression vector for *sspB* | Kan |
| pGA-OX15-int-tw | Non-replicative vector for integrase expression | Amp |
