# Supplementary material for: Demethylmenaquinone Methyl Transferase Is a Membrane Domain-Associated Protein Essential for Menaquinone Homeostasis in Mycobacterium smegmatis
Source: Front Microbiol. 2018 Dec 18;9:3145. doi: 10.3389/fmicb.2018.03145 (PMC6305584; doi:10.3389/fmicb.2018.03145)
Supplement: Supplementary file 2 [file Table_2.docx]

**Table S2**. Oligonucleotides used for PCR amplification.

| **Plasmid** | **Gene** | **Forward Primer**  **Reversed Primer** | |
| --- | --- | --- | --- |
| pMUM039 | *menG* | A185 | atccataTGAGTCGAGCGAGCTTGGAGAAGA |
|  |  | A186 | actGGCGGGCTTGGTGG |
| pMUM040 | *menA* | A166 | tcatccccgatccggaggaatcacttccatATGGCCAGTTTCGCGCAGT |
|  |  | A167 | actGTGGAACGCCAGCGGGAT |
| pMUM055 | Upstream *menG* | A243 | ttttttttcacagagtgGAATTCCGAGAATTCACACG |
|  |  | A244 | ttttttttcacaatgtgGCTCGCTCGACTCACGACG |
|  | Downstream *menG* | A245 | ttttttttccagattttggCACCAAGCCCGCCTAG |
|  |  | A246 | ttttttttccaacttttggGTCTCAAACCGATCCGCGAAA |
| pMUM058 | mTurquoise | A241 | ccagtactaatattGTTAGTAAAAGAGAAGAAC |
|  |  | A242 | CTCGTCGGCTTCGAGTGC |
| pMUM103 | *menJ* | A459 | ttttttttcatatgATGAACACCCGAGCGGATGTG |
|  |  | A460 | actGCTGAACGGCACCCGCTGAT |
| N/A | *menG* KO | A312 | CGGGCTTGCCGATTTTCG |
|  |  | A313 | CGGGTTACCAACCTCTGGTTA |
| pMUM106 | P766-8G promoter | A442 | gctagttaactacgtcgacatcgatttcccttaagattagatatcccgacaattgGCTGCTACCAGGCCTAGATCTG |
|  |  | A443 | ccccatggtaccagaaagcttGGTGGTGCATGCGGTTGT |
|  | *menG* | A444 | aagctttctggtaccatggGGACAGAAAGGAGGAAGGAAT |
|  |  | A445 | aagtcgtcgccaccaatccccatatgctcgagtcgcgaattaatGACTGATAGTGACCTGTTCGTTG |
| pMUM110 | TetR38 | A487 | ggggaaacttaagAGCTGGCTAGCGAGTCATGAGGT |
|  |  | A488 | cccccccgatatcAATATTGGATCACGCCGCGAG |
| pMUM119 | *menG-HA* | A185 | atccataTGAGTCGAGCGAGCTTGGAGAAGA |
|  |  | A506 | tttttcccATTAATTCAGCTGGCGTCCGCGTAGTTCTCGGAGTAGTTCTCGTCGTTGGCGGCCGCGTAGTCCGGGACGTC |
