# Supplementary material for: Demethylmenaquinone Methyl Transferase Is a Membrane Domain-Associated Protein Essential for Menaquinone Homeostasis in Mycobacterium smegmatis
Source: Front Microbiol. 2018 Dec 18;9:3145. doi: 10.3389/fmicb.2018.03145 (PMC6305584; doi:10.3389/fmicb.2018.03145)
Supplement: Supplementary file 3 [file Data_Sheet_1.PDF]

Figure S1

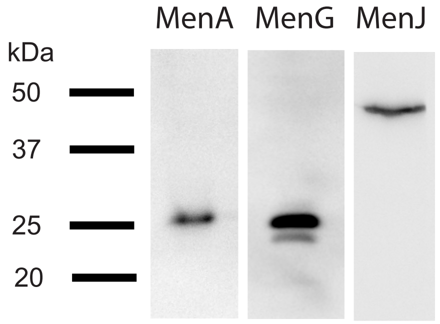

Figure S1. Expression of epitope-tagged MenA-HA (30 kDa), MenG-HA (25 kDa) and MenJ-HA (44 kDa), detected with anti-HA antibody.
