# Supplementary material for: Demethylmenaquinone Methyl Transferase Is a Membrane Domain-Associated Protein Essential for Menaquinone Homeostasis in Mycobacterium smegmatis
Source: Front Microbiol. 2018 Dec 18;9:3145. doi: 10.3389/fmicb.2018.03145 (PMC6305584; doi:10.3389/fmicb.2018.03145)
Supplement: Supplementary file 4 [file Data_Sheet_2.PDF]

Figure S2

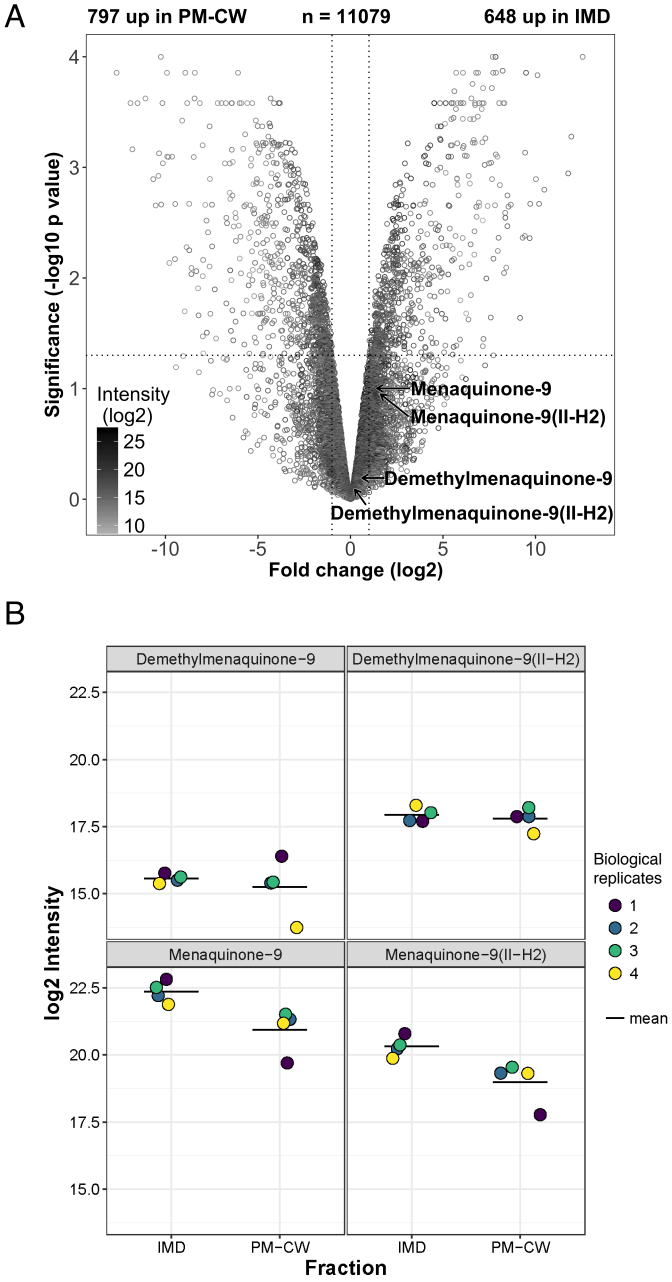

A
797 up in PM-CW
n = 11079
648 up in IMD
Significance (-log10 p value)
Fold change (log2)
Intensity (log2)
Menaquinone-9
Menaquinone-9(II-H2)
Demethylmenaquinone-9
Demethylmenaquinone-9(II-H2)
B
Demethylmenaquinone−9
Demethylmenaquinone−9(II−H2)
Menaquinone−9
Menaquinone−9(II−H2)
log2 Intensity
Fraction
IMD
PM−CW
Biological replicates
1
2
3
4
mean

Figure S2

C

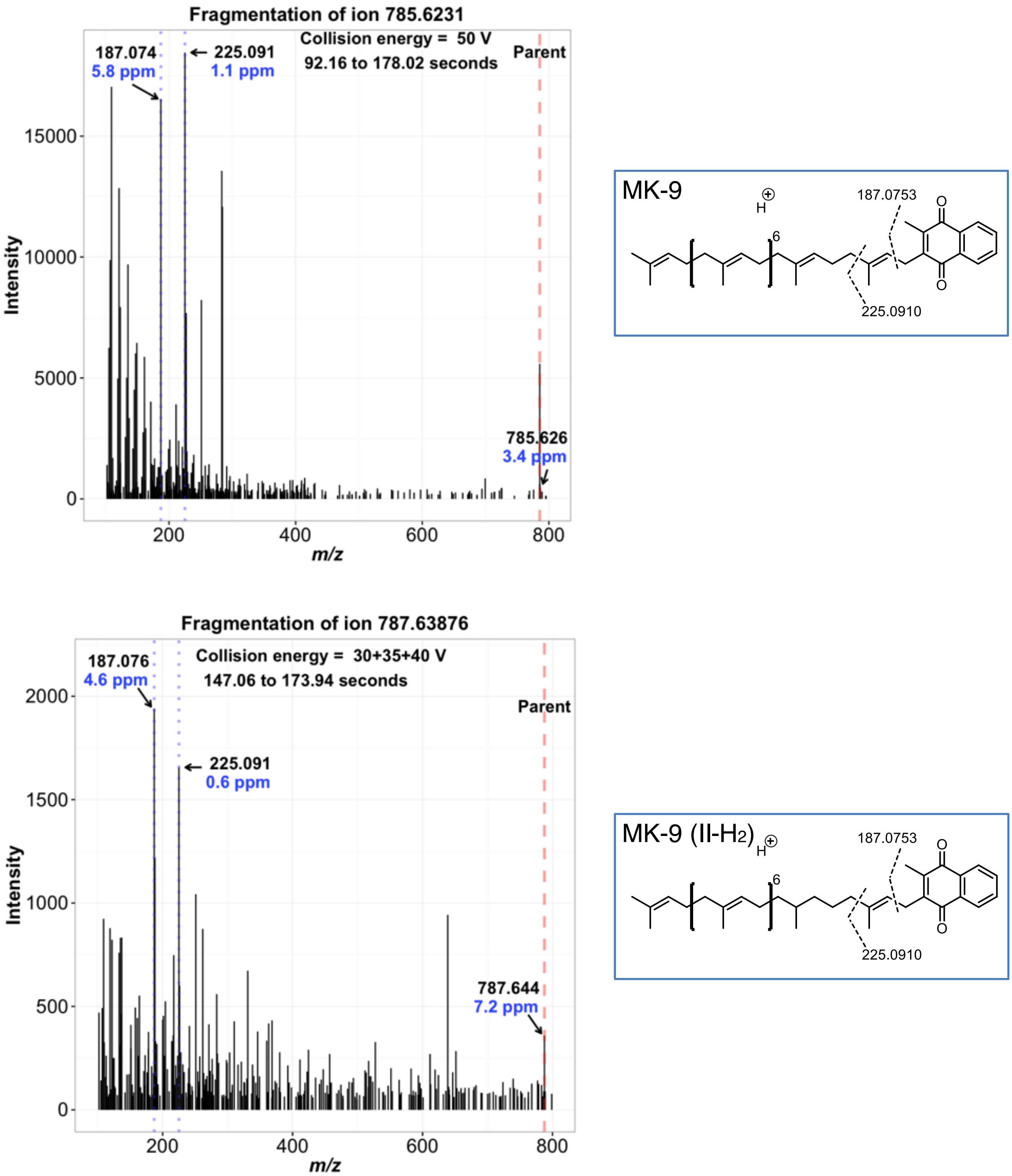

Figure S2. The subcellular distribution of menaquinone species. (**A**) Volcano plot of ions detected after lipidomic profiling of IMD and PM-CW by HPLC-MS. The major menaquinone species, MK-9 (II-H_2) and MK-9 are slightly enriched in the IMD. DMK-9 (II-H_2) and DMK-9 are found more evenly distributed between the IMD and PM-CW. IMD, intracellular membrane domain. PM-CW, plasma membrane associated with cell wall. MK, menaquinone. DMK, demethylmenaquinone. (**B**) Intensity plot of MK-9 species between the IMD and PM-CW. DMK-9 and DMK-9 (II-H_2) are equally present in both fractions and MK-9 and MK-9 (II-H_2) species are slightly enriched in the IMD. (**C**) Collision-induced dissociation mass spectroscopy for MK-9 and MK-9 (II-H_2).
