# Supplementary material for: Demethylmenaquinone Methyl Transferase Is a Membrane Domain-Associated Protein Essential for Menaquinone Homeostasis in Mycobacterium smegmatis
Source: Front Microbiol. 2018 Dec 18;9:3145. doi: 10.3389/fmicb.2018.03145 (PMC6305584; doi:10.3389/fmicb.2018.03145)
Supplement: Supplementary file 5 [file Data_Sheet_3.PDF]

Figure S3

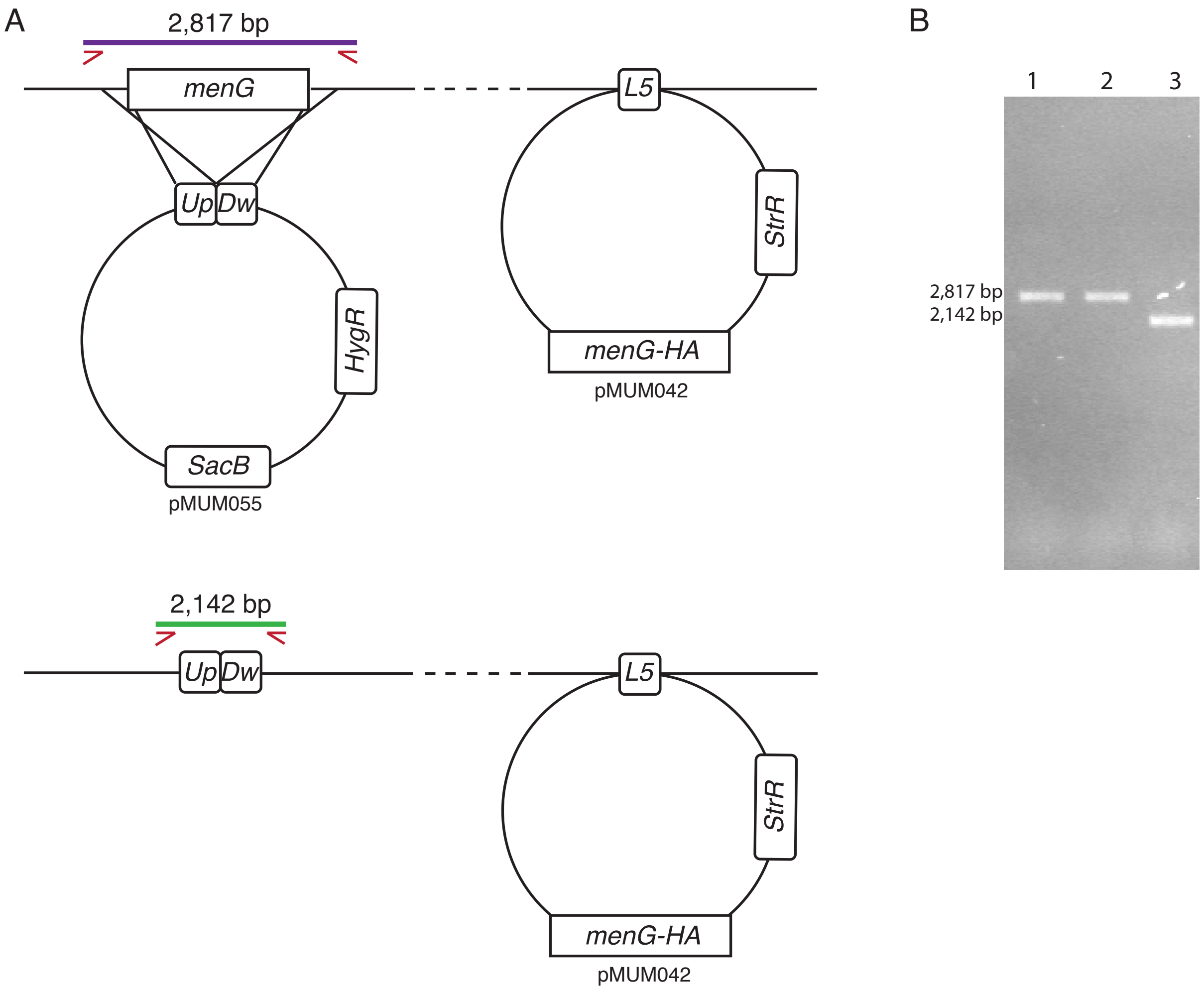

Figure S3. Endogenous *menG* deletion is only possible with extra *menG* copy. (**A**) Scheme for endogenous *menG* deletion. Homologous recombination of pMUM055 upstream and downstream of the *menG* gene in the presence of another copy of the gene, expressed from pMUM042 at the integration site L5. PCR products of *menG* gene (purple) and deletion (green). Final construct, *ΔmenG::menG-HA* Str^R (**B**) PCR products of wild-type (WT) and knockout candidates. Lane 1: WT; Lane 2 WT revertant; Lane 3: *menG* deletion.
