# Supplementary material for: Demethylmenaquinone Methyl Transferase Is a Membrane Domain-Associated Protein Essential for Menaquinone Homeostasis in Mycobacterium smegmatis
Source: Front Microbiol. 2018 Dec 18;9:3145. doi: 10.3389/fmicb.2018.03145 (PMC6305584; doi:10.3389/fmicb.2018.03145)
Supplement: Supplementary file 6 [file Data_Sheet_4.PDF]

Figure S4

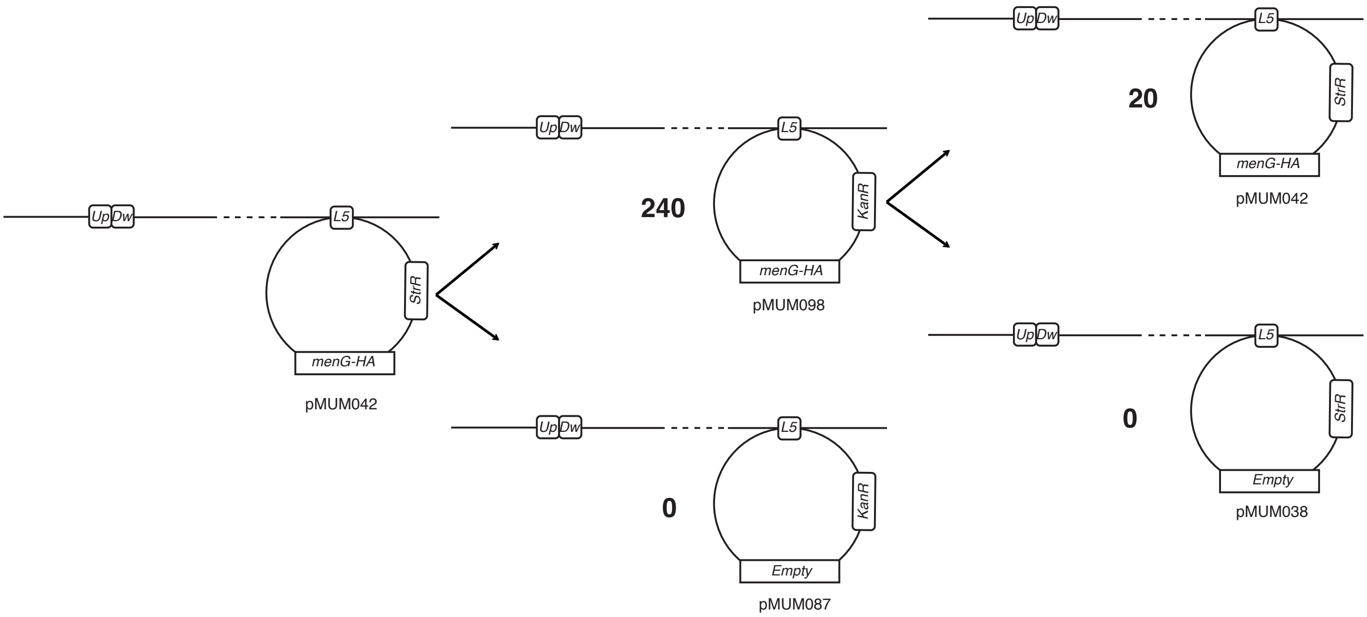

Figure S4. Plasmid swap in a *ΔmenG* background is only possible with MenG expression vectors. Electroporation of pMUM098 (*ΔmenG::menG-HA Kan*^{R}) into *ΔmenG::menG-HA Str*^{R} resulted in plasmid swap, with 240 colonies. Electroporation of pMUM087 (*ΔmenG::empty Kan*^{R}) resulted in no colonies. Electroporation of pMUM042 (*ΔmenG::menG-HA Str*^{R}) into *ΔmenG::menG-HA Kan*^{R} resulted in plasmid swap, while the empty vector pMUM038 (*ΔmenG::empty Str*^{R}) failed to generate colonies.
