# Supplementary material for: Demethylmenaquinone Methyl Transferase Is a Membrane Domain-Associated Protein Essential for Menaquinone Homeostasis in Mycobacterium smegmatis
Source: Front Microbiol. 2018 Dec 18;9:3145. doi: 10.3389/fmicb.2018.03145 (PMC6305584; doi:10.3389/fmicb.2018.03145)
Supplement: Supplementary file 7 [file Data_Sheet_5.PDF]

Figure S5

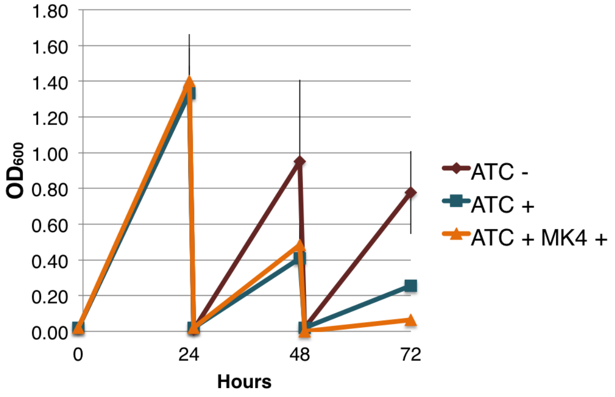


**Figure S5**. MK-4 does not rescue MenG depletion. Growth curve of *ΔmenG::tet*$_{off}$ *menG-DAS::tet*$_{on}$ *sspB* exposed to ATC with and without MK-4 supplementation over 72 h of sub-culturing every 24 h.
