# Supplementary material for: Demethylmenaquinone Methyl Transferase Is a Membrane Domain-Associated Protein Essential for Menaquinone Homeostasis in Mycobacterium smegmatis
Source: Front Microbiol. 2018 Dec 18;9:3145. doi: 10.3389/fmicb.2018.03145 (PMC6305584; doi:10.3389/fmicb.2018.03145)
Supplement: Supplementary file 8 [file Data_Sheet_6.PDF]

Figure S6

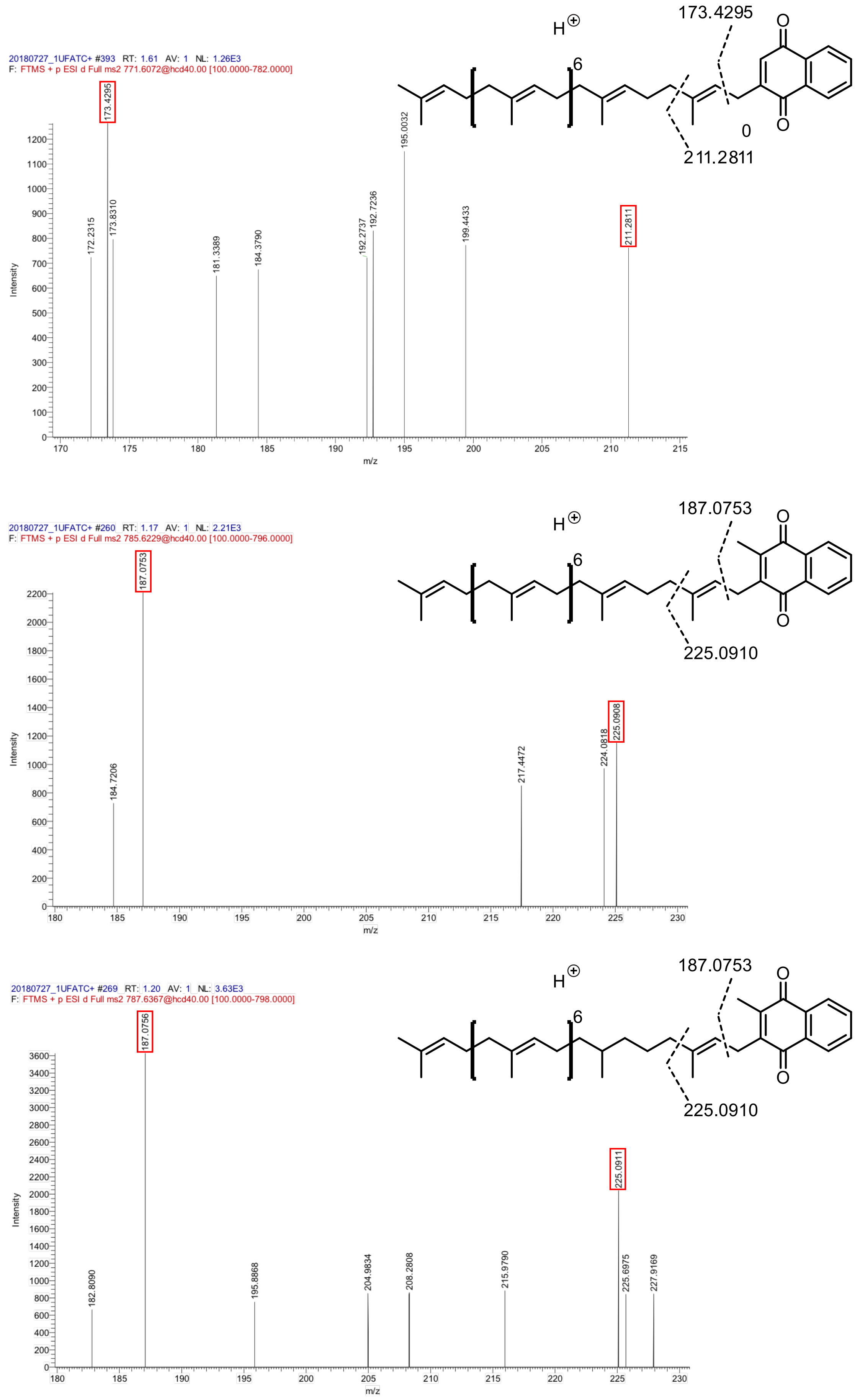

Figure S6. MK species detection confirmation. Representative fragmentation of parental ions DMK-9 *m/z* 771.6075, MK-9 *m/z* 785.6231, and MK-9 (II-H_2) *m/z* 787.6388. Both signature fragments 173.4295 and 211.2811 are present after DMK-9 fragmentation, and 187.0753 and 225.0910 after MK-9 and MK-9 (II-H_2) fragmentation.
