# Supplementary material for: Demethylmenaquinone Methyl Transferase Is a Membrane Domain-Associated Protein Essential for Menaquinone Homeostasis in Mycobacterium smegmatis
Source: Front Microbiol. 2018 Dec 18;9:3145. doi: 10.3389/fmicb.2018.03145 (PMC6305584; doi:10.3389/fmicb.2018.03145)
Supplement: Supplementary file 9 [file Data_Sheet_7.PDF]

Figure S7

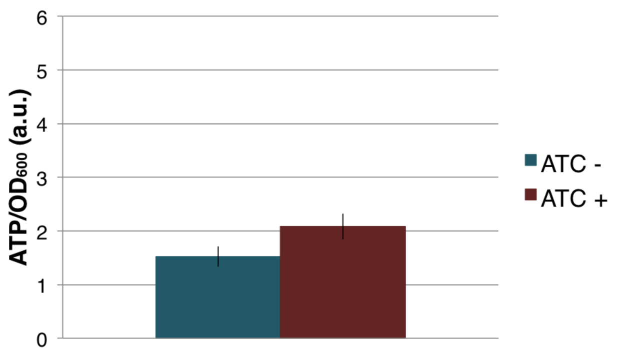

Figure S7. ATP accumulation in *WT::tet$_{on}$ sspB.* ATP accumulation measured over a 24-hour period with and without ATC inducing only expression of SspB. ATC, anhydrotetracycline.
